# Evolution of *Hox* gene clusters in deuterostomes

**DOI:** 10.1186/1471-213X-13-26

**Published:** 2013-07-02

**Authors:** Juan Pascual-Anaya, Salvatore D’Aniello, Shigeru Kuratani, Jordi Garcia-Fernàndez

**Affiliations:** 1 Laboratory for Evolutionary Morphology, RIKEN Center for Developmental Biology, 2-2-3 Minatojima-minami, Chuo-ku, Kobe, Japan; 2 Cellular and Developmental Biology, Stazione Zoologica Anton Dohrn, Villa Comunale, Naples 80121, Italy; 3 Departament de Genètica and Institut de Biomedicina (IBUB), Universitat de Barcelona, Av. Diagonal, 643, Barcelona 08028, Spain

## Abstract

*Hox* genes, with their similar roles in animals as evolutionarily distant as humans and flies, have fascinated biologists since their discovery nearly 30 years ago. During the last two decades, reports on *Hox* genes from a still growing number of eumetazoan species have increased our knowledge on the *Hox* gene contents of a wide range of animal groups. In this review, we summarize the current *Hox* inventory among deuterostomes, not only in the well-known teleosts and tetrapods, but also in the earlier vertebrate and invertebrate groups. We draw an updated picture of the ancestral repertoires of the different lineages, a sort of “genome *Hox* bar-code” for most clades. This scenario allows us to infer differential gene or cluster losses and gains that occurred during deuterostome evolution, which might be causally linked to the morphological changes that led to these widely diverse animal taxa. Finally, we focus on the challenging family of posterior *Hox* genes, which probably originated through independent tandem duplication events at the origin of each of the ambulacrarian, cephalochordate and vertebrate/urochordate lineages.

## Background

*Hox* genes comprise a wide subfamily of homeobox-containing transcription factors. In most eumetazoans studied so far, *Hox* genes are clustered in the same genomic region and are transcribed in the same orientation, although there are cases where the cluster has been split, as in the fruit fly *Drosophila melanogaster*, or has been completely disintegrated, as in the tunicate larvacean *Oikopleura dioica*. Usually, invertebrates possess a single *Hox* cluster, whereas vertebrates possess multiple clusters as a result of several rounds of whole-genome duplications (WGD). Namely, two rounds (2R) of WGD occurred in early vertebrate evolution ([1,2]; see [3] for a review), resulting in the four *Hox* clusters of jawed vertebrates (the so-called HoxA, B, C and D clusters) [4,5]. Teleost fishes experienced an additional third round (3R) of WGD [6-8] resulting in up to seven or eight *Hox* clusters [4,5]. Therefore, the single cluster of invertebrates is thought to be reminiscent of the pre-duplicative state [2]. Within vertebrates, each *Hox* gene can be assigned by sequence comparison to one of 14 different cognate or paralogous groups (PGs) and each cluster retains a subset of these paralogues [9].

The expression patterns of *Hox* genes reflect their position in the cluster: genes at the 3′ end are expressed in and pattern the most anterior part of the embryo, while the genes at the 5′ end pattern more posterior body parts. This phenomenon is known as spatial colinearity. In some animals, like amphioxus (a cephalochordate) and vertebrates, the position in the cluster also determines the onset of expression, with the 3′ genes expressed earlier than the 5′ ones. This phenomenon is called temporal colinearity. As a result of spatial and temporal colinearity, the *Hox* genes are eventually expressed in a nested manner along the main anterior–posterior axis of the animal body, resulting in a *Hox* code that bestows differential structural identity [10].

It is thought that changes in the *Hox* code might be causative for evolutionary novelties, such as the fin-to-limb transition [11,12], the number of vertebrae [13,14], the snake body plan [15] or the presence or absence of ribs in the trunk [16], to mention some examples. To fully understand the nature of the changes in the *Hox* code leading to morphological evolution, it is essential to know the *Hox* content of a wide range of animals belonging to different phyla. Nonetheless, although the *Hox* genes and clusters are relatively well characterized in most groups of vertebrates (mainly in osteichthyans: the bony vertebrates), unfortunately few groups of invertebrates have received similar attention. Importantly, our knowledge of both the *Hox* content and regulation in invertebrate deuterostomes has grown significantly in recent years. In this review, we summarize old and new data and present a detailed picture of the current catalogue of *Hox* clusters in deuterostomes, inferring when possible the putative *Hox* repertoire of the last common ancestor (LCA) condition for different groups. We also discuss the implications of the different *Hox* gene/cluster repertoires within the frame of deuterostome evolution, giving special attention to the posterior genes, which might have originated by independent lineage-specific expansions in ambulacrarians, cephalochordates and vertebrates.

## *Hox* content in invertebrate deuterostomes

**Figure 1:**
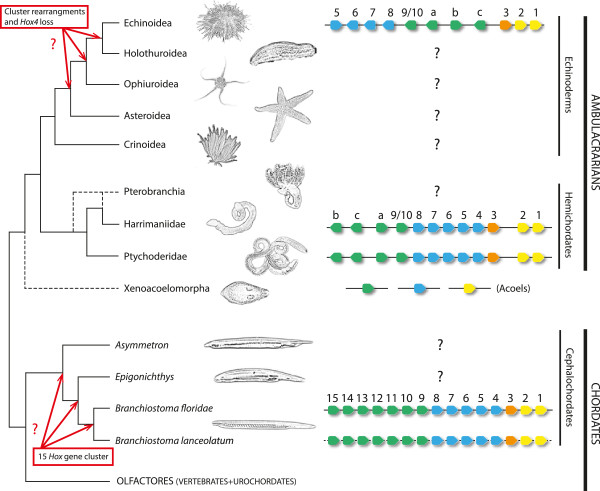
**General phylogenetic tree of deuterostomes showing the *Hox *clusters of non-olfactores deuterostomes known to date.** The *Hox* repertoire of a substantial number of groups within the invertebrate deuterostomes is still lacking (black question marks), and the origin of a 15 *Hox* gene cluster in cephalochordates, or when the *Hox4* was lost in echinoderms are still a mystery (indicated by red question marks and arrows). Yellow, anterior *Hox* genes; orange, *Hox3*; blue, central *Hox* genes; green, posterior *Hox* genes.

Deuterostomes and protostomes are the two major groups of bilaterian animals. Deuterostomes classically consist of two main groups: chordates and ambulacrarians (Figure 1), although recent phylogenetic studies also include the Xenoturbellida and/or Aceolomorpha as deuterostomes (see below). Chordates include cephalochordates (amphioxus), urochordates and vertebrates, the two latter forming the group called Olfactores [17] (Figure 1). The Ambulacraria encompasses a wide group of animals with different morphologies and consists basically of echinoderms (sea urchins, starfish, ophiurids, crinoids and sea cucumbers) and hemichordates (acorn worms and the class Pterobranchia) (Figure 1).

### Xenoturbellida and aceolomorpha: to be or not to be … deuterostomes

Xenoturbellids and acoelomorphs (acoels plus nemertodermatids) were classically classified as platyhelminthes. However, in the last decade and with the advance of molecular phylogenomics, their position has changed dramatically, depending on the methods and datasets used. Whereas acoelomorphs were classified as basal bilaterians [18-21] and Xenoturbellida formed a new phylum within deuterostomes [22,23], the most recent analyses have grouped *Xenoturbella* and acoelomorphs in a monophyletic group, the so-called Xenacoelomorpha [24], either at the base of the Bilateria [25] or as a sister group of Ambulacraria within the deuterostomes [24]. Although the definite positions of xenoturbellids and acoelomorphs remain uncertain, here we will discuss the implications of the different possibilities.

Xenoturbellid *Hox* genes have been studied solely by a polymerase chain reaction (PCR) amplification survey in the species *Xenoturbella bocki*[26]. This study identified only an anterior *Hox1*, three central *Hox* genes (*HoxM1*, *HoxM2* and *HoxM3*) and a posterior *HoxP* gene [26]. Acoels, on the other hand, have only three *Hox* genes: one anterior, one central and one posterior [27,28]. Although acoel *Hox* genes are not clustered [28], they retain a spatially colinear expression pattern (we use the term colinearity with regards to PG order, regardless of gene clustering) [28,29]. Regarding Nemertodermatida, only one study has been reported in which two central and one posterior *Hox* genes were identified by means of degenerate PCR [30].

Establishing a plausible evolutionary scenario for the *Hox* content of the LCA of Xenacoelomorpha remains difficult, because most of the sequences of the above-mentioned PCR surveys are very short and thus the datasets are poor. The simplest landscape is that of a single cluster consisting of one anterior, one central and one posterior *Hox* gene. If the Xenacoelomorpha is indeed confirmed as a group of the Deuterostomata, this would imply—compared with ambulacrarians and chordates—that the lineage suffered a massive loss of *Hox* genes and a complete disintegration of the cluster, at least in acoels. This would be in line with their simplified body plans from a more complex, *Hox*-rich, deuterostome ancestor. If eventually they are classified as the sister group of Nephrozoa (protostomes + deuterostomes), then their *Hox* content would nicely fit with that expected for their earlier bilaterian ancestor.

### Ambulacrarian *Hox* genes

**Figure 2:**
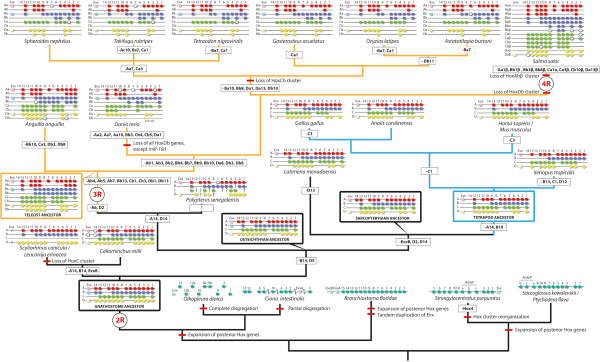
**Reconstructed evolution of *Hox *gene families within deuterostomes.** The *Hox* genes and clusters of those representative species with complete or almost complete *Hox* cluster sequences are shown, and gene losses (thin black squares) or other events (crossed red lines), such as *Hox* cluster duplication or loss are inferred. The ancestral conditions are reconstructed taking into account the information of species with non-complete *Hox* cluster sequences (see the main text). Pre-duplicative clusters are shown in turquoise; vertebrate *Hox* clusters are type-coloured: red, HoxA; blue, HoxB; green, HoxC; yellow, HoxD. For the sake of clarity, the phylogenetic relationships of tetrapods are shown in light blue and those of teleosts in orange. White squares indicate pseudogenes. *Evx* genes are shown when possible, with lighter colours. 2R, 3R and 4R indicate two, three teleost-specific and four salmonid-specific rounds of whole genome duplication, respectively. The phylogenetic relationships of teleosts here are based on [98].

Within ambulacrarian groups, echinoderms are the most well studied, and several PCR surveys have been used to partially determine the *Hox* gene inventories of different species (see [31] and references therein), although the genomic organizations remain unknown in most cases (Figure 1). The exception within echinoderms is the well-known sea urchin *Strongylocentrotus purpuratus*, whose draft genome has been published [32]. *S*. *purpuratus* possesses a single cluster of about 600 kb in length that contains 11 *Hox* genes (Figures 1 and 2). However, the order of the *S*. *purpuratus Hox* genes in the cluster is altered in comparison with other deuterostome *Hox* clusters, so that some anterior genes are near to posterior ones and have different transcriptional orientations (Figures 1 and 2; [33]).

Regarding the hemichordates, the presence of a single *Hox* cluster has been identified recently in two different enteropneust species: *Saccoglossus kowalevskii* and *Ptychodera flava*[34]. Their *Hox* clusters show identical organization, with 12 *Hox* genes arrayed in ~500 kb, all with the same transcriptional orientation except for the terminal pair of Ambulacrarian-specific posterior *Hox* genes *AmbPb* and *AmbPc* (previously named *Hox11*/*13b* and *Hox11*/*13c*, respectively [34]). Overall, the hemichordate *Hox* cluster reflects a more prototypical organization than its sea urchin counterpart and its comparison with that of *S*. *purpuratus* allowed Freeman and colleagues [34] to infer the changes accounting for the scrambled condition of the latter. For example, *S*. *purpuratus* lacks a *Hox* member of the PG4 when compared with hemichordates. Therefore, sea urchins would have lost *Hox4* at some point in echinoderm evolution, probably arising from the genomic rearrangements that provoked first the inversion of *Hox1*–*5* genes and then the translocation of *Hox1*–*4* to the 5′ end of the cluster, eventually causing the loss of *Hox4*[34]. Besides, crinoids (*Metacrinus rotundus*) and asteroids (*Asterina minor* and *Patiriella exigua*) have a *Hox4* gene [35-37]; thus, the loss of *Hox4* must have occurred at least after the split of echinoids, holothuroids, and ophiuroids from the rest of the echinoderms (Figure 1). Nonetheless, the genomic sequences of more ambulacrarian lineages, mainly those within the group of echinoderms, must be investigated to depict a precise evolutionary scenario for the ancestral ambulacrarian *Hox* cluster.

## Cephalochordates

Cephalochordates are the sister group of Olfactores and thus are in a key phylogenetic position to allow the ancestral condition of chordates to be understood. Given the divergent nature of tunicates (see the next section), cephalochordates are also a valuable out-group for evolutionary and comparative studies of vertebrates [38]. The Floridian amphioxus *Branchiostoma floridae* possesses the most prototypical *Hox* cluster identified so far in deuterostomes. It contains 15 *Hox* genes, the largest gene content for a *Hox* cluster hitherto reported, spanning a genomic stretch of ~470 kb and all in the same transcriptional orientation: thus it has not suffered any rearrangements since cephalochordates split from the LCA of chordates more than 500 million years ago (Mya) [39-41]. Similarly, 15 *Hox* genes, presumably in a single cluster, have been described in the European amphioxus *Branchiostoma lanceolatum*, thereby showing that a 15 gene *Hox* cluster is not a species oddity [42]. This discovery indicates that the amphioxus *Hox* cluster probably reflected the ancestral chordate condition, with counterparts for every PG of vertebrates, i.e., that it possessed a complete chordate *Hox* cluster. However, although this holds true for the anterior and medial PGs, it is still a matter of debate for the posterior genes, as phylogenetic trees do not show clear orthologous relationships between posterior genes from different deuterostome phyla. This phenomenon was thought to be the consequence of the higher evolutionary rate of the posterior part of the cluster, the so-called deuterostome posterior flexibility [43]. However, an alternative scenario is that some posterior genes originated independently in the different lineages by tandem duplication events, as claimed by recent reports [34,44-46] that we will discuss below. Nonetheless, to further clarify this topic, genomic information for other cephalochordate genus, such as *Asymmetron* sp., an earlier branch of cephalochordates than *Branchiostoma* and *Epigonichthys* ([47]; Figure 1), might give more unambiguous insights into the ancestral cephalochordate *Hox* cluster condition and eventually into the ancestral chordate *Hox* cluster.

In addition to the doubtful nature of the posterior part of the *Hox* cluster, we recently reported the breaking of colinearity for some amphioxus *Hox* genes, where the most striking case was that of *Hox14*, found to be expressed in the cerebral vesicle [48]. Interestingly, the brains of other animals are characterized as *Hox*-negative regions, making the amphioxus case a surprising oddity and therefore bringing up the possibility that the amphioxus *Hox* cluster and its regulation are not as prototypical as previously thought.

## Urochordates

The *Hox* complements of the urochordate genomes sequenced thus far provide little information of use in inferring the ancestral condition of the Olfactores (and thus the preduplicative state of vertebrates), because their genomes are very divergent as reflected in their body plans, with dramatic genome rearrangements and extensive gene losses.

Among the urochordates, the best-known model is the ascidian *Ciona intestinalis*, which possesses a rather disorganized set of *Hox* genes [49,50]. The *C*. *intestinalis Hox* cluster has experienced a partial disintegration, although there are genomic stretches where several *Hox* genes are linked [49,50]. These groupings are *Hox1*, *2*, *3, 4*, *5, 6* and *10* in chromosome 1 and *Hox12* and *13* in chromosome 7 [51]. On the other hand, *Hox7*, *8*, *9* and *11* are absent in all ascidians sequenced so far [50,52]. Moreover, two independent translocation events disrupted the order of *Hox* genes in chromosome 1: *Hox10* is located between the *Hox4* and *Hox5* genes, and the *Hox*-related gene *EvxA* sits between *Hox1* and *Hox2*. In fact, some authors consider that *sensu stricto*, *Hox1*–*10* genes are not linked because they span ~5 Mb [50], indicating that the ascidian *Hox* repertoire has a partially disintegrated nature. Surprisingly, even with such a disrupted organization, most ascidian *Hox* genes are expressed in a colinear fashion in the central nervous system (CNS) [50]. Another urochordate, the larvacean *O*. *dioica*, shows a dramatic disintegration of the cluster with all the *Hox* genes scattered along the genome. Only two genes—*Hox9a* and *Hox9b*—are linked, probably as a result of a species-specific tandem duplication event [53]. Moreover larvaceans seems to have lost *Hox3*, *5*, *6*, *7* and *8*[53]. Additionally, it has been proposed that the previously named *Hox11* gene of *O*. *dioica* is in fact *Hox12*, something that is consistent between phylogenetic trees and non-phylogenetic tree-based methods. Thus, *O*. *dioica Hox11* and *12* should be renamed as *Hox12a* and *Hox12b* (Figure 2[46]), which implies that also *Hox11* was lost in *O*. *dioica*, as in other ascidians. Strikingly, as in *C*. *intestinalis*, *O*. *dioica Hox* genes were expressed with partial spatial colinearity in the CNS [53].

## Vertebrates

The increased number of *Hox* clusters in vertebrates compared with their invertebrate counterparts illustrates clearly the history of genome duplications. Following the 2R-WGD at the base of the vertebrates, tetrapods retained four clusters, whereas teleost fishes expanded to seven or eight clusters arising from a teleost-specific 3R-WGD [5] and salmonids up to 13 clusters after an additional salmonid-specific 4R-WGD [54,55] (Figure 2). In all WGD events, the duplication of the *Hox* cluster was followed by differential *Hox* gene losses, eventually resulting in a unique combination of *Hox* genes in every group, like a bar code (a “genomic *Hox*-bar code”). Accordingly, it would be possible to determine to which group a genome of unknown source would belong, just by observing the *Hox* gene/cluster content.

In the following sections, we collate what is known about the *Hox* gene families in different vertebrate groups regarding their genomic configuration and plausible evolutionary origins and modifications.

### *Hox* genes of cyclostomes: the main vertebrate gap

Cyclostomes (the only extant group of agnathans or jawless vertebrates) are the sister group of gnathostomes (jawed vertebrates) and are much less known than the latter, also in terms of *Hox* gene content. Cyclostomes are composed of two different groups: lampreys and hagfishes. In the case of the hagfish, the most recent report is that of Stadler *et al*. [56], who obtained up to 33 *Hox* genes using degenerate PCR, a lower number than that of a general gnathostome, but still compatible with multiple clusters. The authors [56] concluded from their analyses that cyclostomes had split at least after the first WGD and that subsequent independent gene/cluster duplications expanded their *Hox* inventory. However, their analysis was based on very short sequences, so their conclusions remained very speculative. A conclusive repertoire of *Hox* genes and clusters of the hagfish is far from definitive, since the genomic organization of the hagfish *Hox* genes still remains a mystery. New advances in hagfish research [57,58] together with the new and powerful sequencing techniques available will surely help to fill this gap in the future.

Regarding lampreys, several PCR surveys have been done using different lamprey species: *Petromyzon marinus*[59-63], *Lampetra planeri*[64] and the Japanese lamprey *Lethenteron japonicum*[44,65,66]. Moreover, the draft genome of the sea lamprey (*P*. *marinus*) has been published recently [67] and the *Hox* genes were investigated. A total of 25 *Hox* genes were found but only two *Hox* clusters were recognizable: clusters Pm1Hox (with *Hox2*, *3*, *4*, *5*, *6*, *7*, *8*, *9* and *11*) and Pm2Hox (*Hox1*, *4*, *5*, *7*, *8*, *9*, *10* and *11*). Besides these clusters, eight additional genes were found lacking genomic information, except for two of them that are linked, indicating the presence of a putative third cluster [67].

Because the information from the above-mentioned studies is rather scattered and incomplete in terms of a definitive lamprey *Hox* repertoire, we have compiled all the information of genes and clusters reported thus far for lampreys in Table 1. Taking into account that there are genes identified exclusively in some individual studies, a total of 44 distinct lamprey *Hox* genes are recognizable (this number can vary, depending on whether some are just allelic variations; see Table 1). These 44 *Hox* genes would represent the ancestral condition of lampreys, and some of them might not be present in all species. Also, we propose a shift and normalization of the nomenclature of lamprey *Hox* genes, using the Greek alphabet (as in [44]) to indicate the absence of a clear homologous relationship to the gnathostome HoxA, B, C and D clusters. Assignments of some lamprey central *Hox* genes to PG5–7 remain doubtful (Table 1). Interestingly, no *Hox* gene belonging to PG12 has been found in any of these studies (Table 1), suggesting the possible loss of all *Hox12* genes in the LCA of lampreys.

**Table 1:** **Predicted orthology relationships by sequence comparison of all *Hox * genes reported to date in different lamprey species to infer the condition of the LCA of lampreys**

**PG**	**Lamprey Hox Gene**	***Petromyzon marinus***				***Lethenteron japonicum***	***Lampetra planeri***	**GenBank Acc. No**
		**Pendleton *et al. ***[59]	**Amores *et al. ***[60]** / Force *et al. ***[62]	**Irvine *et al. ***[63]	**Smith *et al. ***[67]	**Takio *et al. ***[65]**,**[66]** / Kuraku *et al. ***[44]	**Sharman *et al. ***[64]	
1	*Hox1α*	Pm27-a					LpHox1A (5 nt different: syn)	L14893; AF044797
	***Hox1β***	Pm6-b*	1w	1B	Pm2Hox1w (5 nt, 3 aa different; 3 nt del.)	LjHox1w	LpHox1B (Identical)	L14902; AF434665; AB286671; AF044798
	*Hox1y*	Pm87-c			PmHox1 (1 nt, 1 aa different)			L14908; ENSPMAT00000011284 (Ensembl)
	*Hox1δ*	Pm62-d*					LpHox1C (4 nt different: syn)	L14904; AF044799
2	***Hox2α***	Pm6-e		E2	Pm1Hox2			L14890; AF410908; JQ706314
	*Hox2β*					LjHox2 (5 nt different from E2)	LpHox2A (Identical to LjHox2)	AY497314; AF044800
3***	***Hox3α***			3	Pm1Hox3	LjHox3d		AF410909; AB125270; JQ706315
	*Hox3β*						LpHox3A	AF044801
4	***Hox4α***	Pm33-n**	4w		Pm1Hox4w	LjHox4w (1nt different from 4w)	LpHox4-7B (1 nt different from 4w)	L14896; AF434666; AB125269; AF044803; JQ706316
	***Hox4β***				Pm2Hox4			JQ706323
	*Hox4γ*	Pm2-i	4x			LjHox4x	LpHox4-7E (3 nt, 1 aa different)	L14891; AY056469; AB125278; AF044806
	*Hox4δ*	Pm99-g	4y	G4				L14912; AF410911
	*Hox4ϵ*	Pm88-h						L14909
5-7***	***Hox5α***			N5	Pm1Hox5 (1nt, 1aa different)			AF410915; JQ706317
	***Hox6α***	Pm33-n**	6w (1nt different: syn)	N6	Pm1Hox6	LjHox6w (4nt different from N6/Hox6: syn)		L14896; AF071235; AF410916; AB125275; JQ706318
	***Hox7α***			N7	Pm1Hox7			AF410917; JQ706319
	***Hox5β***	Pm63-l	51	L5/6	Pm2Hox5			L14905; AF410914; JQ706324
	***Hox7β***	Pm4-k	83	K6/7	Pm2Hox7	LjHox6/7m (3 nt different from K6/7: syn)	LpHox4-7C (1 nt different from LjHox6/7m: syn)	L14897; AF410913; AB125272; AF044804
	*Hox5-7γ*	Pm22-f	31	F5/6/7	PmHox7		LpHox4-7D (3 nt different: syn)	L14892; AF410910; AF044805; ENSPMAT00000011116 (Ensembl)
	*Hox5-7δ*	Pm54.T7m						L14899
	*Hox5-7ϵ*	Pm66-j	5w	J5/6/7		LjHox5w (1 nt different: syn)	LpHox4-7A (Identical to LjHox5w)	L14906; AF071234; AF410912; AB125277; AF044802
	*Hox5-7ζ*	Pm74-o					LjHox5i (2 nt different)	L14907; AB125276
	*Hox5-7η*	Pm50-p						L14898
8	***Hox8α***	Pm57-q		Q8	Pm1Hox8Q (4 nt, 1 aa different; 6 nt del.)	LjHoxQ8 (4nt different from Hox8: syn)		L14901; AH005896; AB125274; JQ706320
	***Hox8β***			Q8a	Pm2Hox8Qb			AF035589; JQ706325
	*Hox8γ*	Pm60-r		R8	PmHox8 (2 nt different: syn)		LpHox8A (3 nt different: syn)	L14903; AF035588; AF044807; ENSPMAT00000005057 (Ensembl)
	*Hox8δ*					LjHox8p (7 nt, 2 aa different from Q8a)		AB125273
9	***Hox9α***	Pm28-v	9y	V9	Pm1Hox9 (6 nt, 2 aa different)			L14889; AF410919; JQ706321
	***Hox9β***	Pm29-t	9w	T9	Pm2Hox9 (1 nt different: syn)	LjHox9r (5 nt different from T9: syn)	LpHox9B (4 nt different from T9: syn)	L14894; AF410918; AB125271; AF044810; JQ706326
	*Hox9γ*	Pm94-u	9x				LpHox9C (3 nt different: syn)	L14910; AF044811
	*Hox9δ*	Pm98-s			PmHox9 (Scaffold_6175)		LpHox9A (2 nt different: syn)	L14911; AF044809; ENSPMAT00000011060
	*Hox9ϵ*				PmHox9 (Scaffold_16685)			ENSPMAT00000011449 (Ensembl)
10	*Hox10α*			W10b		LjHox10s (5 nt different from W10b: syn)		AF410921; AB286673
	***Hox10β***	P3-w	10w	W10a	Pm2Hoxa10b	LjHoxW10a (4nt different from W10a: syn)	LpHox10B (2 nt different from W10a: syn)	L14895; AF410920; AB286672; AF044813
	*Hox10γ*	Pm56-x		X10				L14900; AF410922
	*Hox10δ*						LpHox10A (5 nt, 1 aa different from X10)	AF044812
11	***Hox11α***	***		Z11a	Pm1Hox11 (1nt, 1aa different)			AF410924; JQ706322
	***Hox11β***				Pm2Hox11a			ENSPMAT00000010946 (Ensembl)
	*Hox11γ*	***	11w	Y11				AF410923
	*Hox11δ*			Z11b				AF410925
	*Hox11ϵ*					LjHox11t		AB286674
13	*Hox13α*					LjHox13α	LpHox13A	AB293597; AF044814
	*Hox13β*	PmHox13β				LjHox13β		AB293598; ENSPMAT00000000840 (Ensembl)
14	*Hox14α*					LjHox14α		AB293599

* Just 2 nucleotides between Pm6-b and Pm62-d, but non-synonymous: probably two different genes. They also have Lampetra counterparts.

** Clone n of Pendleton *et al.*[59] corresponded to two different genes in Force *et al.*[62]: Hox4w and Hox6w.

*** Clone 139 and Hox11 clones mentioned in [63] as personal communication by W.J. Bailey, and *Hox3y* and *Hox5x* from [62], are not taken into account for lacking a published sequence.

Note: LpHox8B (AF044808) sequence from reference [64] contains two undetermined nucleotides, N, and could be orthologue of any other Hox8 gene previously identified.

Note 2: In the case of *Pm2Hox1w* the whole coding sequence is available to compare, and that is why is more polymorphic than other cases, whith shorter sequences to align.

Genes for which there are linkage data are shown in bold. Bold α and β genes belong to Pm1Hox and Pm2Hox clusters, respectively. See [67] and [68].

nt, nucleotides; aa, amino acids; syn, synonymous; del, deletion.

The number of *Hox* clusters of the lamprey can be estimated to be four or five: there are four cognates for at least PG1, 8 and 10 (see Table 1); strikingly, there are five distinct genes for PG4, 9 and 11 and although there are four *Hox1* genes, the cluster Pm1Hox does not seem to have a *Hox1* member [67]. Conversely, there might be fewer clusters, depending on allelic polymorphisms or independent tandem duplication events within a cluster [63]. Nonetheless, none of the above-mentioned scenarios is conclusive and further investigation is needed. At present, it is not clear whether lampreys hold representatives of the four gnathostome *Hox* clusters or whether some originated by independent duplication events (for example, if five clusters are confirmed) [68]. Finally, phylogenetic analysis of dozens of gene families [69] and the recent analysis of the *P*. *marinus* genome [67] pointed to a post 2R-WGD origin of cyclostomes, indicating that the LCA of vertebrates most probably possessed four *Hox* clusters. Accordingly, the synteny of non-*Hox* genes linked to the *Hox* clusters has been generally conserved between cyclostomes and gnathostomes, although with a differential retention of paralogues [67].

### Condrichthyans: a *Hox* cluster loss

The gnathostomes consist of two main groups: condrichthyans (cartilaginous fishes) and osteichthyans (bony vertebrates); and all of them possess members of the four *Hox* clusters (A, B, C and D), indicating that they diverged after the 2R-WGDs [70]. The condrichthyans are divided into two subclasses: Elasmobranchii (sharks, skates and rays) and Holocephali (chimaeras).

Apart from some partial reports on the horn shark *Heterodontus francisci* (elasmobranch) [71-73], the first complete condrichthyan *Hox* repertoire was reported in a chimaera, the elephant shark *Callorhinchus milii* (holocephalian). *C. milii* possesses a total of 45 *Hox* genes, retaining the *HoxD14* gene, plus two *Hox14* pseudogenes (A and B, see Figure 2). The *C*. *milii Hox* repertoire substantially increased the putative *Hox* contents of the gnathostome ancestor [74]. Surprisingly, elasmobranchs seem to have lost the HoxC cluster completely. Studies with both the lesser-spotted cat shark *Scyliorhinus canicula* and the little skate *Leucoraja erinacea* did not find any of the HoxC cluster members, including sequences encoding *Hox* cluster-associated microRNAs [75,76]. These are the first reports on the loss of a complete *Hox* cluster type (A, B, C or D) within jawed vertebrates, a loss that dates back to 250 Mya [77].

### Osteichthyans

The *Hox* clusters of osteichthyans are the most well-known within vertebrates and deuterostomes. Here we include recent reports on newly investigated species that have changed the overall scenario of the previously inferred ancestral conditions [5].

Osteichthyans comprise two major groups: sarcopterygians (coelacanths, lungfish and tetrapods) and actinopterygians (ray-finned fishes, including teleosts). Within the former, the complete repertoire of many tetrapods (chicken, mouse and human genomes among others) is known [5,78]. Comparison of the *Hox* inventories of different tetrapods allows to infer a tetrapod ancestral condition of up to 41 genes [78], one more than previously thought [5], and an amniote ancestral condition of 40 *Hox* genes (after the loss of *HoxC1*; Figure 2), of which only the green anole (*Anolis carolinensis*) retains all of them [78,79], while mammals and the chicken have lost the *HoxC3* gene independently. Liang *et al*. [78] also did not identify *HoxC3* in crocodiles and turtles by degenerate PCR and it is absent from turtle genomes [80,81]. Therefore, *HoxC3* was probably lost before the evolutionary split of archosaurians and turtles. Amphibian repertoires vary: *Xenopus tropicalis* has 38 *Hox* genes, three fewer than in the ancestral tetrapod. One of these lost genes is *HoxC1*, also lost from amniotes. Because *HoxC1* is still present in the amphibian caecilian *Ichthyophis bannanicus*[78], it means that it was lost independently in amniotes and *X*. *tropicalis* (Figure 2). Taking into account data from *I*. *bannanicus* and the salamander *Batrachuperus tibetanus*[78], the amphibian ancestor probably had 40 genes, after losing *HoxD12* from its tetrapod ancestor (Figure 2).

The *Hox* clusters of the coelacanths *Latimeria menadoensis*[82] and *Latimeria chalumnae*[83], the closest living relative to the sarcopterygian ancestor [83], include a *HoxA14* gene that was later lost in tetrapods. Moreover, a PCR survey of the lungfish *Protopterus annectens* also found a *HoxA14* gene ([78]; data from the lungfish are not included in Figure 2 because they lack clustering information, but they were taken into account to infer the ancestral condition). This enabled Liang and colleagues [78] to reconstruct a more complete ancestral sarcopterygian *Hox* inventory, with a total of 43 *Hox* genes (Figure 2).

The case of actinopterygians is rather more complicated, because after the teleost-specific 3R-WGD, more genes—and in some cases a complete cluster—were lost differentially (Figure 2). Teleosts are the most numerous group of vertebrates with more than 27,000 extant species [84]. It has been already 15 years since more than four *Hox* clusters were found in the cyprinid zebrafish *Danio rerio*[60,85], which instead has seven *Hox* clusters: HoxAa, HoxAb, HoxBa, HoxBb, HoxCa, HoxCb and HoxDa. The cluster HoxDb has been reduced to only the *Hox*-related microRNA *miR*-*10d*[86]. Another cyprinid, *Megalobrama amblycephala*, might also lack the HoxDb cluster [87], implying that it was probably lost in the cyprinid ancestor. Other teleosts with known *Hox* complement include pufferfishes, cichlids, medakas, sticklebacks, salmonids and some early lineages such as the eel and the bichir (although data are not complete for the last species). The *Hox* complement of several pufferfishes has been already reported: *Spheroides nephelus*[88], *Takifugu rubripes*[88,89] and *Tetraodon nigroviridis*[90] showing differential species-specific losses (see Figure 2). The case of *T*. *rubripes* is curious. Amores and colleagues described a possible duplication of the *HoxAa* cluster in *T*. *rubripes*, which they called *HoxAc*[88]. This cluster duplication was considered doubtful by other authors [4,5]. Later it was found that the sequence of this *HoxAc* cluster, present in version 2.0 of the genome of *T*. *rubripes*, corresponded to a bacterial artificial chromosome (BAC) clone of the Nile tilapia and was not present in later versions of the *T*. *rubripes* genome [91]. Comparisons of the *Hox* complement of the above-mentioned pufferfishes with those of the medaka *Oryzia latipes*[92], cichlids such as the Nile tilapia *Oreochromis niloticus*[93] (not present in Figure 2 because the clusters are largely not sequenced), of *Astatotilapia burtoni*[94,95] and the three-spine stickleback, *Gasterosteus aculeatus*[94], show a common loss of the HoxCb cluster in their LCA (the ancestral Neoteleosteii; see Figure 2). Moreover, independent losses of *Hox* genes are not rare: for example *HoxBa7* and *HoxCa1*[94,95] have been lost several times (Figure 2). *HoxBa7* is present in *O*. *niloticus* and thus was independently lost in the medaka and *A*. *burtoni*.

Strikingly, the Atlantic salmon *Salmo salar* and the rainbow trout *Oncorhynchus mykiss* contain 13 *Hox* clusters [55,96,97], arising from a salmonid-specific 4R-WGD [54], the former with a total of 118 *Hox* genes plus eight pseudogenes: the largest *Hox* repertoire to date [55]. *S*. *salar* lost the HoxDb cluster before the 4R-WGD (as did zebrafish independently). *S*. *salar* still retains *HoxD1a* copies (α and β) within the HoxDa cluster. Therefore, *HoxD1a* was indeed present in the teleost ancestor, in contrast to previous ideas [5]. The complete repertoire of *Hox* clusters of the European eel *Anguilla anguilla*, representing the earliest branch of teleosts [98], helped in reconstructing a more reliable teleost ancestral inventory [99]. *A*. *anguilla* also retains *HoxD1a*, like *S*. *salar*, meaning that it was independently lost later in zebrafish and neoteleosts (e.g., medakas, pufferfishes, cichlids and sticklebacks; Figure 2). *A*. *anguilla* is the best representative of the teleost ancestor, having lost only four *Hox* genes (three of them are pseudogenes) and retaining all eight *Hox* clusters after the 3R-WGD ([99]; Figure 2). The osteoglossomorph *Hiodon alosoides* (the goldeye), a representative of a basal branch diverging after the eels [98], also seems to have retained the original eight clusters [100]. In summary, the teleost ancestor probably had at least 74 *Hox* genes, with eight *Hox* clusters (after the 3R-WGD), quickly followed by differential gene and cluster losses in the different lineages ([99]; Figure 2).

There are few reports on *Hox* genes of fishes outside the teleosts. Several studies report either few *Hox* genes or partial information about *Hox* clusters for the Senegal bichir *Polypterus senegalus*. The bichir is a representative of the earliest branch of actinopterygians that diverged prior to the 3R-WGD. Only the HoxA cluster sequence out of a total of four clusters has been reported thus far [101,102]. There is also a report of cDNA screening in another bichir species, *Polypterus palmas*[103]. Although again, while the data presented are fragmented and not complete, these studies allow to infer the presence of *HoxD2* in the LCA of osteichthyans (and actinopterygians), a gene that was lost in all the osteichthyans species studied so far except in the bichir ([102]; Figure 2). In another gnathostome species, the catshark *S*. *canicula*, *HoxD2* is expressed in very important tissues such as the pharyngeal arches and the rhombencephalon [104]. However, we predict that the loss of this gene may have little importance in the general patterning of such tissues, because the expression of *Hox* genes is quite redundant; once a gene is lost, other *Hox* genes patterning the pharynx and the hindbrain could compensate for its function. The information for other early branches of actinopterygians (the paddlefish, sturgeon, gar and *Amia calva*), diverging after the bichir, is also scattered and partial [6,105], although it is important to note that some of them, for instance sturgeon and paddlefish, probably have more than four *Hox* clusters caused by lineage-specific WGDs [105]. In fact, the American paddlefish *Polyodon spathula* has at least two HoxA and two HoxD clusters (each termed α and β, to denote their different duplicative origins from the teleost 3R-WGD). Surprisingly, *P*. *spathula* retains a *HoxD14β* paralogue [105], indicating that, unlike as previously inferred, *HoxD14* was present in the LCA of actinopterygians (and thus in the osteichthyan LCA) and was secondarily lost in the rest of the lineages of the group (shown in Figure 2).

## The unsolved origin of the deuterostome posterior *Hox* genes

More than a decade ago, the discovery and analysis of the posterior *Hox* genes in amphioxus reflected the problematic issue of their one-to-one orthology assignments with their vertebrate counterparts [43]. This phenomenon, also present in posterior ambulacrarian *Hox* genes (the posterior AmbP genes, namely *Hox11*/*13a*, *b* and *c*, form an independent clade [34]), was explained by a possibly higher rate of evolution of the posterior *Hox* genes, eventually precluding their grouping in phylogenetic trees. This process was called deuterostome posterior flexibility [43]. The discovery of the PG14 in vertebrates [73], disallowed alternative explanations because it equated the number of vertebrate PGs to the number of amphioxus *Hox* genes known by that time and because of the amount of tandem duplications and losses implicated in an origin by independent duplications. Therefore, the notion of deuterostome posterior flexibility was commonly accepted. However, in the last few years, new results based on different methods have turned the tables. We will try here to integrate the information from both phylogenetic trees performed previously by several studies [34,41,44,46] and from two methods not based on phylogenetic trees [45,46] and summarize them into the most parsimonious scenario.

**Figure 3:**
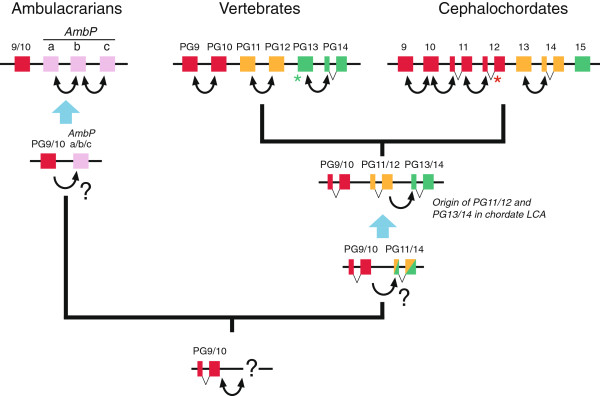
**Possible evolutionary scenario for the origin of posterior *Hox *genes in amphioxus, ambulacrarians and vertebrates.** The presence of the second intron splitting the homeobox into two exons is shown in those genes where it is present, and inferred in ancestral conditions. The red asterisk indicates that the amphioxus *Hox12* gene intron is in a different position and thus was acquired secondarily. The green asterisk indicates that *Hox13β* of the lamprey *L. japonicum* has retained the ancestral intron, but that intron has been lost in all other vertebrate *Hox13* genes. The black question marks indicate the unclear evolutionary origin of *AmbP* and PG11/14 ancestral genes: they could have been originated independently, or were present in the last common ancestor of deuterostomes. The existence of more *Hox* genes in the ancestral states cannot be excluded at this time.

It is remarkable that amphioxus *Hox9*–*12* genes seldom group in a one-to-one relationship with their vertebrate cognates, but they tend to group together in a single clade. The same occurs with amphioxus *Hox13*–*15*[34,41,46]. Interestingly, the amphioxus *Hox9*–*12* and *Hox13*–*15* clades usually group with vertebrate *Hox9*–*10* and *Hox11*–*14*, respectively, suggesting an independent origin for these genes from at least two ancestral posterior *Hox* genes, as proposed in one of the evolutionary scenarios put forward by Freeman *et al*. [34]. However, how vertebrates and amphioxus obtained their final set of posterior *Hox* genes is trickier, so we have pictured a likely evolutionary scenario (represented in Figure 3), based on several lines of evidence. First, the recently discovered amphioxus *Hox15* gene groups with vertebrate PG13 in a well-supported clade [34,41] and both of them group with vertebrate PG14. In addition, a weight matrix-based method also assigned amphioxus *Hox15* to PG13 [46]. This would imply the presence of at least one ancestral PG13/14 gene in chordates from which the amphioxus *Hox15* and vertebrate PG13 and PG14 genes originated (Figure 3; green boxes). Alternatively, amphioxus may have lost the PG14 cognate secondarily if there were both PG13 and PG14 genes ancestrally. Second, amphioxus *Hox13* and *Hox14* generally group together, suggesting an origin by tandem duplication in the cephalochordate lineage. In addition, the amphioxus Hox13/14 clade tends to fall in a bigger clade with vertebrate PG11–12, implying a common origin (an ancestral PG11/12 gene: Figure 3; orange boxes). Third, as mentioned above, amphioxus *Hox9*–*12* genes tend to group together in an independent clade [41], suggesting their independent origin in this taxon by a tandem duplication event. Moreover, amphioxus *Hox9*–*12* genes tend to fall within the vertebrate PG9–10 clade, symptomatic of the existence of an ancestral PG9/10 gene in chordates (Figure 3; red boxes). Therefore, the last common ancestor of chordates had at least three posterior *Hox* genes (see Figure 3): one PG9/10, one PG11/12, from which amphioxus *Hox13*–*14* originated and one PG13/14, from which amphioxus *Hox15* originated. The two latter probably come from a PG11/14 ancestral gene, because all chordate *Hox11*–*15* genes form a monophyletic clade [34], implying a first condition of two ancestral genes, that quickly expanded into a three-gene condition arising from a duplication in tandem (Figure 3).

When including the available data from ambulacrarians to this hypothesis, it becomes still more complicated. On one side, the ambulacrarian *Hox9*/*10* genes group with their chordate cognates PG9 and PG10, also proved by non-phylogenetic tree-based methods [45,46], suggesting that the LCA of deuterostomes likely had the PG9/10 ancestral gene. On the other hand, the phylogenetic relationships between ambulacrarian *AmbP* genes and the chordate PG11–14 genes are not consistent between different studies and several scenarios are possible (see [34]). Thomas-Chollier *et al*. [46] assigned *AmbP* genes to the vertebrate PG9, suggesting an independent origin of these genes from the ancestral PG9/10 gene in the ambulacrarian lineage (Figure 3), but, because there are no other studies suggesting this, the origin of *AmbP* remains unsolved.

This entire evolutionary scenario gets even more complicated when considering the presence of introns within the homeobox. *Hox* genes have generally only one intron, splitting the gene into two exons, the second one containing the homeobox. However, some posterior *Hox* genes possess a second intron splitting the homeobox into two exons. These are the vertebrate *Hox14* genes, lamprey *Hox13β* and amphioxus *Hox11*, *Hox12* and *Hox14*. This second intron is in the same position for all of them (except for amphioxus *Hox12*) and equal to the second intron position of the dipteran posterior *Abdominal*-*B* genes [44,73]. Common introns suggest an ancestral origin, because it seems more probable to gain an intron once and then lose it secondarily, than to gain it in the same position independently [106]. Therefore, and within the evolutionary frame suggested above, ambulacrarian posterior *Hox* genes lost this intron in the LCA of ambulacrarians, while in vertebrates, the two or three ancestral posterior *Hox* genes contained this intron, which subsequently was lost in different genes independently in the amphioxus and vertebrate lineages; finally amphioxus *Hox12* gained secondarily a different second intron (Figure 3).

## Review and conclusion

In summary, the posterior *Hox* genes are thus more flexible than central and anterior *Hox* genes, at least in terms of non-stasis: thus, while the number of posterior *Hox* genes seems to have changed independently in the different deuterostome lineages (Figure 3), the numbers of anterior and central *Hox* genes have been kept fixed since the deuterostome LCA (PG1–8) [34]. These more recent changes in the posterior part of the cluster may also explain why the non-coding regions of this part of the cluster are less conserved than those of more anterior parts [42,74,107]. This new *cis*-deuterostome posterior flexibility implies a lack of regulatory constraints for the PG9–14 cognates that eventually allowed posterior genes to be uncoupled from the stricter *Hox* code of more anterior genes [44,48,108] or to be co-opted to pattern novel morphological structures, such as the limbs and genital tracts of vertebrates.

### Future perspectives

We have presented here a catalogue and a current view on the evolution of *Hox* gene families in deuterostomes, showing that we are still far from picturing a conclusive scenario for the ancestral conditions. In fact, many early branches within vertebrates remain to be examined, as do more invertebrate deuterostomes (for example, more ambulacrarians); hence we cannot exclude the existence of more *Hox* genes in the ancestral states until the number of sequenced genomes from non-model animals has increased considerably. With the new genome projects being carried out currently in a wide range of animals, mainly in vertebrates (e.g., the 10 K Genome Project [109]), this landscape will change in the very near future. On the other hand, the problematic origin of the posterior *Hox* genes will not be solved just by reporting more *Hox* genes, but also will require improved methods, both phylogenetic and non-phylogenetic.

## Competing interests

The authors declare that they have no competing interests.

## Authors’ contributions

JPA and JGF conceived of the study. JPA, SDA and JGF drafted the manuscript. SK critically revised and discussed the study. All authors read and approved the final manuscript.
